# Increased prevalence of autoantibodies neutralizing IFNλ2/3 in young individuals with cystic fibrosis

**DOI:** 10.70962/jhi.20250268

**Published:** 2026-01-12

**Authors:** Kevin Groen, Chau Tran, Roger Kuratli, Marie E. Egan, Johannes Trück, Emanuela M. Bruscia, Marc Emmenegger, Benjamin G. Hale

**Affiliations:** 1 Institute of Medical Virology, University of Zurich, Zürich, Switzerland; 2 Department of Pediatrics, Yale University School of Medicine, New Haven, CT, USA; 3 Department of Cellular and Molecular Physiology, Yale University School of Medicine, New Haven, CT, USA; 4 Divisions of Allergy and Immunology, and the Children’s Research Center, University Children’s Hospital Zurich, University of Zurich, Zürich, Switzerland; 5 Division of Medical Immunology, Department of Laboratory Medicine, University Hospital Basel, Basel, Switzerland

## Abstract

Neutralizing interferon-λ2/3 autoantibodies are identified in 11.8% of individuals with cystic fibrosis, >10-fold the prevalence in controls. Autoantibody-containing plasmas enhance respiratory virus replication in vitro and associate with allergic rhinitis in patients, suggesting that interferon-λ2/3 autoantibodies may impact mucosal pathology.

## Teaser

Groen et al. identify neutralizing interferon-λ2/3 autoantibodies in 11.8% of individuals with cystic fibrosis, a greater than 10-fold higher prevalence than in control individuals. Autoantibody-containing plasmas enhanced respiratory virus replication in vitro, and associated with allergic rhinitis in patients, suggesting that interferon-λ2/3 autoantibodies have the potential to impact mucosal pathology.

Human type I, II, and III interferons (IFNs) are cytokine components of innate immunity against pathogens. IFN-Is (e.g., IFNα and IFNβ) and IFN-IIIs (IFNλ1, IFNλ2, and IFNλ3) are most critical for immediate defenses against viruses, while IFN-II (IFNγ) acts predominantly against mycobacteria and fungi (1). Though receptors for IFN-Is and IFN-II are ubiquitous, IFN-III receptors are mainly localized to mucosal barriers in respiratory and gastrointestinal epithelia, restricting IFN-III function anatomically (1). Autoantibodies (autoAbs) neutralizing IFN-Is or IFN-II exacerbate severity of many viral or mycobacterial infections, respectively. Nevertheless, there is no well-characterized association of anti-IFN-III autoAbs with any disease (2). Previous evidence suggests that IFN-III polypeptides may be recognized by plasma IgG antibodies at an increased frequency in persons with cystic fibrosis (pwCF) (3, *Preprint*). Cystic fibrosis (CF) is a rare monogenic disorder leading to abnormal mucosal secretions that can promote chronic respiratory bacterial infections and increase susceptibility to acute viral infections (4). Here, we aimed to survey and functionally characterize neutralizing anti-IFN-III autoAbs in a CF cohort. We identify neutralizing IFNλ2/3 autoAbs in 11.8% of pwCF, a >10-fold higher prevalence than in controls. AutoAb-containing plasmas enhanced respiratory virus replication in vitro, and were associated with allergic rhinitis in patients, suggesting their potential to impact mucosal pathology in pwCF.

**Figure 1.**
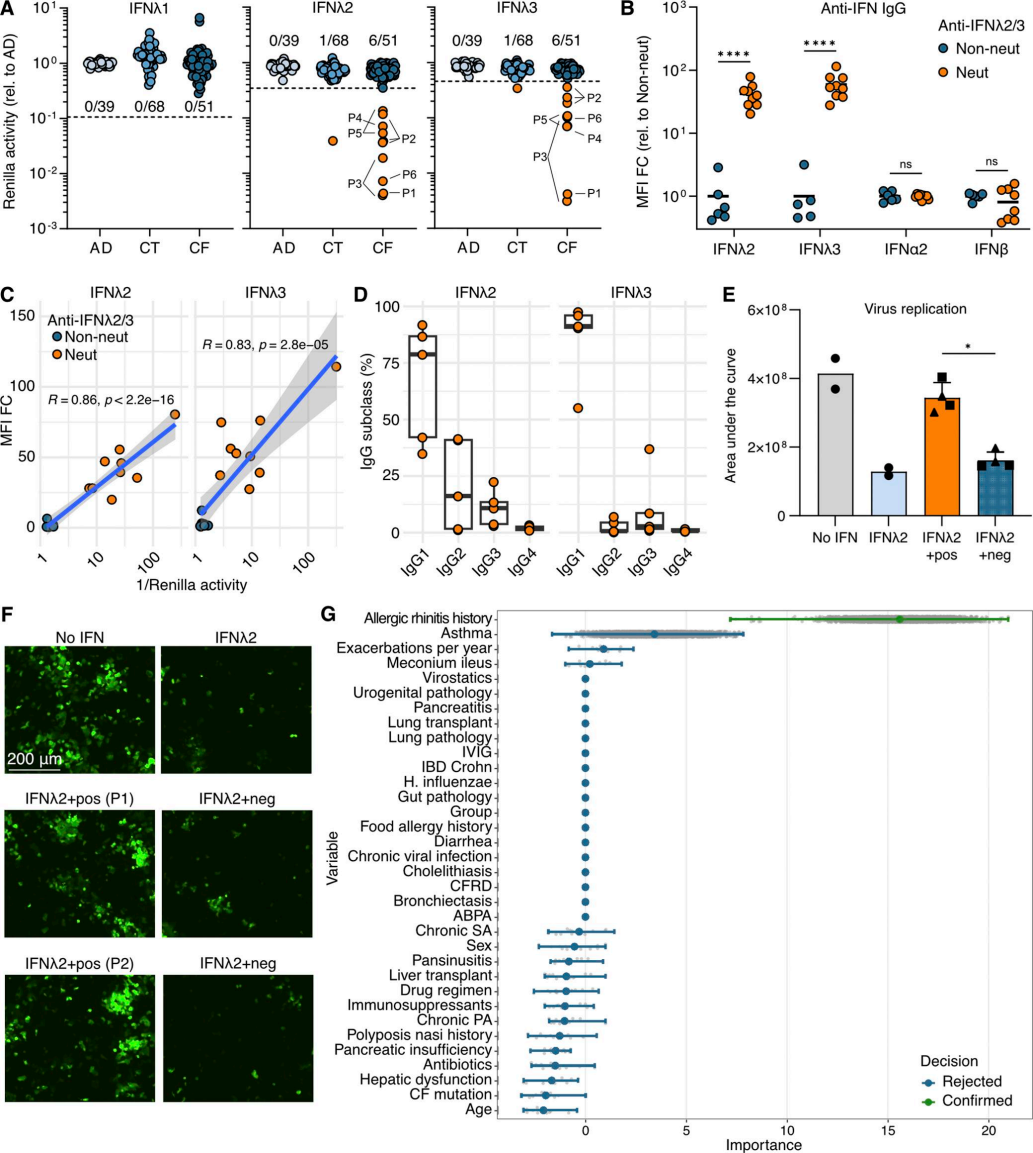
**At least 10% of young individuals with CF harbor neutralizing anti-IFNλ2/3 IgG autoAbs. (A)** IFNλ1/2/3 neutralization by plasmas from individuals with AD (*n* = 39), various adults (CT; *n* = 68), or pwCF (CF; *n* = 84 samples from *n* = 51 individuals). Dotted lines indicate thresholds set using standard outlier calculations. Neutralizing plasmas are orange; numbers represent unique individuals; positive individuals are labeled. **(B)** Anti-IFN-III and anti-IFN-I IgGs in CF plasmas positive (neut; *n* = 9) or negative (non-neut; *n* = 6) for IFNλ2/3 neutralization. **(C)** Spearman’s correlation between IFNλ2/3 neutralization (A) and IgG (B). **(D)** Anti-IFNλ2/3 IgG subclasses in CF plasmas (*n* = 5). **(E)** Area under the curve from PIV5-GFP replication kinetics in A549 cells pretreated with IFNλ2 (or not) in the presence of pwCF plasmas positive (pos) or negative (neg) for IFNλ2/3 neutralization. Values are triplicate means from two experiments. Error bars indicate standard deviations. For the IFNλ2+pos and IFNλ2+neg groups, data are from two patients (squares/triangles) from two experiments. **(F)** Fluorescence images of infected cells from E. **(G)** Random forest regression of clinical variables in pwCF positive (*n* = 4) or negative (*n* = 16) for anti-IFNλ2/3 autoAbs. Dots represent importance scores per iteration (maximum 5,000); error bars reflect importance spread. Abbreviations: MFI FC, median fluorescence intensity fold-change; IVIG, intravenous immunoglobulin; IBD, inflammatory bowel disease; CFRD, CF-related diabetes; ABPA, allergic bronchopulmonary aspergillosis; SA, *Staphylococcus aureus*; PA, *Pseudomonas aeruginosa*. Statistical analyses were two-way ANOVA with the Šidák correction (B), Spearman correlation (C), and Mann–Whitney test (E) (ns, nonsignificant; *P < 0.05; ****P < 0.0001).

We tested 84 plasmas from 51 young pwCF (median age 12.4 years, interquartile range [IQR] 8.7–18.9 years, 43.1% female) for their ability to neutralize 10 ng of IFNλ1, IFNλ2, or IFNλ3 at a 1:100 plasma dilution. Sample availability limited testing of lower plasma dilutions that could have increased detection sensitivity. We similarly screened plasmas from 39 young individuals with respiratory or food allergies (allergic disease [AD]; median age 11.4 years, IQR 8.6–13.9 years, 46.2% female), and 68 older individuals with various non–CF-related diseases (see Materials and methods: control [CT]; median age 64.0 years, IQR 51.2–77.0 years, 47.0% female). While IFNλ1 neutralization was not observed by any plasma, IFNλ2/3 neutralization was detected in 6/51 individuals (11.8%; 10/84 samples) of the pwCF group, 0/39 individuals (0%) of the AD group, and 1/68 individuals (1.5%) of the CT group (Fig. 1 A). IFNλ2 and IFNλ3 are 96% identical, which likely accounts for their similar autoreactivity profiles between individuals. Multiple longitudinal samples were available for three positive CF group individuals, all of which had IFNλ2/3-neutralizing activity (Fig. 1 A). The overall observed 0.9% IFNλ2/3 neutralization prevalence in the combined AD/CT groups (1/107) is broadly similar to that previously observed in a larger mixed group of 1,489 individuals (median age 66 years, IQR 48–84 years, 57% female) (2), and is substantially lower (>10-fold) than the 11.8% prevalence in pwCF described here. The six pwCF (four males and two females) with plasmas neutralizing IFNλ2/3 had a median age of 19.6 years when first identified (IQR 15.9–22.0 years; with the youngest being 8 years old).

We then compared anti-IFN IgG binding in plasmas neutralizing, or not, IFNλ2/3. Neutralizing plasmas had significantly higher levels of anti-IFNλ2/3 IgG than the subset of non-neutralizing plasmas tested (Fig. 1 B). The IgG responses were specific to IFN-III, as anti-IFNα2/β IgG levels were negative in the IFNλ2/3-neutralizing plasmas (Fig. 1 B), which is consistent with previous high-throughput profiling of the pwCF cohort that found enrichment of IgG autoreactivity toward IFN-IIIs but not IFN-Is (3, *Preprint*). Furthermore, anti-IFNλ2/3 IgG levels strongly correlated with IFNλ2/3-neutralizing activity (Fig. 1 C). IgG subtyping of anti-IFNλ2/3 IgGs showed that the largest proportion of anti-IFNλ2/3 IgGs were IgG1 (Fig. 1 D).

IFN-IIIs limit virus replication, protect epithelial barriers, and suppress allergic airway disease (1). We therefore investigated whether plasmas with neutralizing anti-IFNλ2/3 IgGs impact antiviral effects in vitro. A549 cells were pretreated with 10 ng/ml IFNλ2 alone, or in combination with pwCF plasmas that had (or not) neutralizing anti-IFNλ2/3 IgGs. Pretreatment of human lung epithelial cells with IFNλ2 attenuated replication of the respiratory virus, parainfluenza virus 5 (PIV5) (Fig. 1, E and F). Over multiple experiments, two independent pwCF plasmas containing neutralizing anti-IFNλ2/3 autoAbs negated the IFNλ2 antiviral effect and enhanced PIV5 replication (Fig. 1, E and F). Importantly, two independent plasmas from pwCF negative for anti-IFNλ2/3 autoAbs could not inhibit IFNλ2 action (Fig. 1, E and F).

We next explored whether any clinical variables might associate with neutralizing anti-IFNλ2/3 autoAbs in pwCF by comparing data from four pwCF positive for anti-IFNλ2/3 autoAbs and 16 pwCF negative for anti-IFNλ2/3 autoAbs (data from others were not available). In this small subcohort, 4/4 (100%) individuals with neutralizing anti-IFNλ2/3 autoAbs experienced a history of allergic rhinitis compared to 1/16 (6%) without anti-IFNλ2/3 autoAbs. Random forest regression analysis confirmed the possible association between neutralizing anti-IFNλ2/3 autoAbs in pwCF and allergic rhinitis, while other typical markers of CF disease remained unassociated (Fig. 1 G).

In summary, neutralizing anti-IFN-III autoAbs were observed in 11.8% of young pwCF, a >10-fold enrichment over that in other cohorts (here and [2]). These autoAbs are primarily IgG1, limit IFN-III antiviral activity in vitro, and appear to be directed toward IFNλ2/3, not IFNλ1. Although our study is not powered to draw definitive conclusions, it is tempting to speculate that IFNλ2/3 neutralization in pwCF might contribute to more severe impairment of mucosal barrier function (where IFN-III receptors are primarily localized), a hallmark of CF (4), or exacerbated infectious/allergic airway disease symptoms. Future studies in different and larger CF cohorts will be required to dissect such consequences, as well as to define the genetic and immunological factors promoting anti-IFNλ2/3 autoAb development in subsets of pwCF. It is possible that the unique mucosal environment of pwCF, who frequently experience infections with respiratory pathogens, could create a chronically inflamed setting that increases the likelihood of anti-IFN-III autoAb onset. Our findings lay a foundation to explore new roles for IFN-IIIs, and anti-IFN-III autoAbs, in CF pathogenesis.

## Materials and methods

### Human plasma samples and ethics

Plasma samples from individuals with respiratory allergy (pollen allergy and/or asthma) or food allergy (AD group; *n* = 39 patients/samples) were obtained at the University Children’s Hospital Zurich, Zürich, Switzerland. Plasma samples from individuals with other various diseases (including acute or postacute COVID-19, multiple myeloma, chronic lymphocytic leukemia, chronic myelomonocytic leukemia, melanoma, small cell lung cancer, Lynch syndrome, Guillain–Barré syndrome, or HIV) were collected at the University Hospital Zurich, Zürich, Switzerland, originally for routine laboratory analyses (CT group; *n* = 68 patients/samples). We made use of these two cohorts with approval of the Kantonale Ethikkommission Zürich (BASEC IDs: 2018–01042, 2020–01731, and 2024-00666). Plasma samples from individuals with CF (CF group; *n* = 51 patients; *n* = 84 samples) were obtained from the Yale University School of Medicine, New Haven, CT, USA, during routine clinical visits and stored as part of the Yale CF Pediatrics Research Center biobank, with informed consent obtained in accordance with relevant U.S. laws and institutional guidelines. The collection and study were approved by the Yale University School of Medicine Human Investigation Committee (#0906005332). All research was conducted in accordance with the provisions of the Declaration of Helsinki and the Good Clinical Practice guidelines of the International Conference on Harmonization. Written informed consent was received prior to participation.

### Quantitative detection of anti-IFN-I/III IgGs and IFN-III–neutralizing activity

Detection of anti-IFN-I/III IgG autoAbs in patient plasmas was performed with a multiplexed, bead-based assay using 1:100 diluted plasma samples, similar to described (5). Protein targets were: IFNλ1 (NBP2-34996; Novusbio), IFNλ2 (8417-IL; R&D Systems), IFNλ3 (5259-IL; R&D Systems), IFNα2 (NBP2-34971, Novusbio), and IFNβ (11420-1; PBL Assay Science). IgG subclasses were determined using subclass-specific secondary antibodies. Detection of neutralizing IFN-III activity was performed in A549-Interferon-Reporter cells similar to described previously using 1:100 diluted plasma samples (5). Resulting Renilla luciferase activities were determined using a PerkinElmer EnVision (EV2104). Luciferase values were normalized to those of in-house negative controls that were assayed in parallel and were expressed as relative Renilla luciferase activities. Thresholds for determining positive neutralization were set using standard outlier calculations (the 25% quartile minus 1.5× the IQR) of the entire dataset (AD, CT, and CF groups combined) for each IFN-III.

### Virus assays

Replication of GFP-encoding PIV5 (PIV5-GFP; P523; ViraTree) in A549 cells was assayed as described previously (5), assessing the effects of 1:100 diluted plasma samples and 10 ng/ml IFNλ2 (8417-IL; R&D Systems). GFP expression was monitored every 4 h over the course of 60 h using an IncuCyte S3 Live-Cell analysis system (Sartorius). Total green integrated intensity (Green Calibrated Units × µm^2^/image) values were used to calculate area under the curve values.

### Statistics

Statistical analyses were performed using GraphPad Prism 10 software or R 4.4.2. Comparisons were made using tests described in the figure caption. Significance is denoted as: ns, not significant; *P < 0.05; ****P < 0.0001. For clinical associations, a random forest regression to analyze variable importance for a binary outcome (absence or presence of anti-IFN-III autoAbs) was conducted with the Boruta package. A maximum of 5,000 iterations was employed.

## Data Availability

The data are available from the corresponding authors upon reasonable request.
